# Subseasonal relationship between Arctic and Eurasian surface air temperature

**DOI:** 10.1038/s41598-021-83486-5

**Published:** 2021-02-18

**Authors:** Hye-Jin Kim, Seok-Woo Son, Woosok Moon, Jong-Seong Kug, Jaeyoung Hwang

**Affiliations:** 1 School of Earth and Environmental Sciences, Seoul National University, Gwanak-ro, Gwanak-gu, Seoul, 08826 South Korea; 2 Department of Mathematics, Stockholm University, Stockholm, Sweden; 3 Nordic Institute for Theoretical Physics (NORDITA), Stockholm, Sweden; 4 Division of Environmental Science and Engineering, Pohang University of Science and Technology (POSTECH), Pohang, Korea

**Keywords:** Climate sciences, Environmental sciences

## Abstract

The subseasonal relationship between Arctic and Eurasian surface air temperature (SAT) is re-examined using reanalysis data. Consistent with previous studies, a significant negative correlation is observed in cold season from November to February, but with a local minimum in late December. This relationship is dominated not only by the warm Arctic-cold Eurasia (WACE) pattern, which becomes more frequent during the last two decades, but also by the cold Arctic-warm Eurasia (CAWE) pattern. The budget analyses reveal that both WACE and CAWE patterns are primarily driven by the temperature advection associated with sea level pressure anomaly over the Ural region, partly cancelled by the diabatic heating. It is further found that, although the anticyclonic anomaly of WACE pattern mostly represents the Ural blocking, about 20% of WACE cases are associated with non-blocking high pressure systems. This result indicates that the Ural blocking is not a necessary condition for the WACE pattern, highlighting the importance of transient weather systems in the subseasonal Arctic-Eurasian SAT co-variability.

## Introduction

The warm Arctic-Cold Eurasia (WACE) pattern is well recognized as a dominant interannual climate variability in the boreal winter^1–3^. This seesaw-like surface air temperature (SAT) variability has recently received a significant attention due to the time lag. It was particularly found that the Arctic sea ice reduction and the resulting warm SAT anomaly over the Barents-Kara seas (BKS) often precede cold SAT anomaly over the Eurasia by a few months^4–7^. Such a relationship implies that Arctic climate variability can be used as a potential source for seasonal prediction of Eurasian SAT anomaly^8,9^ especially when combined with North Atlantic sea surface temperature^10^.

The WACE relationship also appears on the subseasonal time scale^11–19^. Kug et al*.*^12^ showed that the low-frequency variability of BKS SAT typically leads Eurasian SAT variability by approximately two weeks. This time lag becomes much shorter if the high-frequency variability is considered. Luo et al*.*^18^, for instance, showed that daily BKS SAT anomaly accompanies Eurasian SAT anomaly with only one-day time lag. This short-term co-variability is not directly induced by Arctic sea ice loss but caused by atmospheric circulation^15,17,19^. A series of studies have shown that daily BKS sea ice change is a response to atmospheric circulation over the Ural region rather than a trigger^13,14,17^.

As a key driver of the subseasonal WACE pattern, the Ural blocking has been highlighted in the literature^15,17,19,20^. A blocking high can effectively generate an anti-correlation between BKS and Eurasian SAT anomalies by modulating the moisture and temperature advection, especially when the background wind or the potential vorticity gradient is weak^15,18^. Physically, the Arctic warm anomaly has been often attributed to the increased downward longwave radiation by the enhanced moisture transport into the Arctic^15,21,22^. Likewise, Eurasian cold anomaly has been related to the reduced downward longwave radiation^15^. However, warm anomaly in the Arctic, resulting from the Ural blocking could immediately increase the upward longwave radiation, resulting in a weak net longwave radiation^23^. In this regard, other studies have suggested the temperature advection as a key driver^1,24,25^. The relative importance of the temperature advection and diabatic heating, however, is not well quantified.

Although the subseasonal WACE pattern has been related to the Ural blocking^15,17,19,20^, it is not clear whether the Ural blocking, which is quasi-stationary or slowly moves westward in time, is a necessary condition. A transient system that travels eastward in time could also generate the WACE pattern. Such possibility, however, has not been addressed in the literature.

In this study, we revisit the daily relationship between Arctic and Eurasian SAT anomalies. Since the relationship could vary from month to month as background flow changes, its subseasonality is first examined. Unlike previous studies which have focused on the WACE pattern, both the WACE pattern and its opposite, the so-called cold Arctic-warm Eurasia (CAWE) pattern, are considered. Although the CAWE pattern is inferred from the mode of interannual SAT variability^1–3^, its spatio-temporal distribution and the driving mechanism have been rarely addressed on the subseasonal time scale.

When examining the WACE pattern, the presence of the Ural blocking is not presumed. Instead the WACE pattern associated with the Ural blocking is compared to that without blocking. The physical processes responsible for the WACE and CAWE patterns are then quantified by computing the temperature budget. The budget analysis reveals the relative importance of the temperature advection against the diabatic heating.

## Data and methods

The six-hourly and daily atmospheric variables, which include SAT, sea level pressure (SLP), geopotential height at 500 hPa (Z500), and 850-hPa horizontal winds (U and V), vertical velocity (ω), and temperature (T) wind (U), are obtained from the European Centre for Medium-Range Weather Forecasts (ECMWF) Re-Analysis-Interim (ERA-Interim)^26^ for the period of 1979–2017. The spatial resolution of these data is 1.5° × 1.5°. The daily anomaly is defined as a deviation from the long-term climatology for each calendar day. The long-term trend is not removed here as its impact is minimal. Although not shown, overall results do not change much when the detrened data are utilized.

To investigate the optimal time lag between the two variables, a time-lagged linear regression analysis is applied:

$$ y\left( {d,t + \tau } \right) = r\left( {d,\tau } \right)x\left( {d,t} \right) + \varepsilon \left( {d,\tau } \right) \left( 1 \right) $$

where $r\left( {d,\tau } \right)$ is a regression coefficient of $y\left( {d,t + \tau } \right)$ with respect to $x\left( {d,t} \right)$ for the selected calendar day $d ( =$ 1 to 365 days) with time lag $\tau ( = -$ 20 to 20 days). The last term, $\varepsilon \left( {d,\tau } \right)$, is the residual. A regression window is 30 days, starting from calendar day $d$. Here the calendar day *d* starts from 1 July 1979 to 30 June 1980 to examine the seasonal variability centered on winter. To keep the same length, the time series, a function of t, is then constructed with the regression windows for 38 years starting from the selected $d$. For instance, if $d$ is 1 July, the time series is constructed by combining the following 30 days (i.e., 1–30 July) from 1979 to 2016 (30 days × 38 years = total of 1140 days). The statistical significance of the regression coefficient is evaluated with the Student’s t-test. The effective number of degrees of freedom is determined by considering the autocorrelation^27^.

The composite analyses are further conducted for WACE and CAWE cases. These cases are first identified by computing the dipole index (DI):

$$ {\text{DI}} = SAT_{BK} - SAT_{Eurasia} \left( 2 \right) $$

where $SAT_{BK}$ and $SAT_{Eurasia}$ are area-averaged SAT anomalies over BKS and Eurasia, respectively. The BKS domain is set to 30°–70°E and 70°–80°N, whereas the Eurasian domain is set to 50°–130°E and 35°–50°N from a daily correlation map (Fig. S1). This definition is similar to the seasonal-mean WACE index used in some previous studies^19,28^. When the WACE pattern is pronounced, DI is expected to be positive. The opposite is true for the CAWE pattern. Both WACE and CAWE cases are defined as a time period when the DI index exceeds one standard deviation for at least three consecutive days. The minimum interval between the DI maxima is set to 15 days to avoid duplicated selections. If multiple DI maxima are detected within 15 days, the largest one is selected as a case. The date of maximum (or minimum) DI index is assigned as the WACE (or CAWE) date, and the time lags from -10 to 10 days are allowed for the case composite. A total of 78 WACE cases (about 2.05 times per year) and 70 CAWE cases (about 1.84 times per year) are identified from November to February (NDJF).

The Arctic-Eurasian SAT (co-)variability is investigated by computing the temperature tendency equation:

3 $$ \frac{\partial T}{{\partial t}} = - V_{H} \cdot \nabla_{H} T + S_{p} \omega + {\text{Q}} + {\text{Res}} $$

where $\partial T/\partial t$ is the temperature tendency, horizontal advection ${ } - V_{H} \cdot \nabla_{H} T$, adiabatic heating, vertical advection $S_{p} \omega$, diabatic heating Q, and residual ${\text{Res}}$. In Eq. (3), $V_{H}$ is the horizontal wind vector, $\omega$ is the pressure velocity, and $S_{p}$ is the stability parameter represented by $S_{p} = \left( {R/C_{p} } \right)\left( {{\text{T}}/p} \right) - \left( {\partial T/\partial p} \right)$ where *R* is the gas constant for dry air (= 287 ${\text{J kg}}^{ - 1} {\text{ K}}^{ - 1}$) and $C_{p}$ is the specific heat at constant pressure. Unlike other variables, Q is derived from the ECMWF forecast. The last term in Eq. (3), Res, includes both the forecast errors and the numerical errors. This tendency equation is basically calculated at 850 hPa. To clarify the mechanism for the temperature change over complex terrain, the same budget analysis is applied at 925 hPa. Each budget term is calculated using six-hourly raw data and averaged into daily. The daily anomalous budget is examined, instead of the raw daily budget, in order to quantify the contribution of individual terms to the temporal evolution of temperature tendency.

## Results

**Figure 1:**
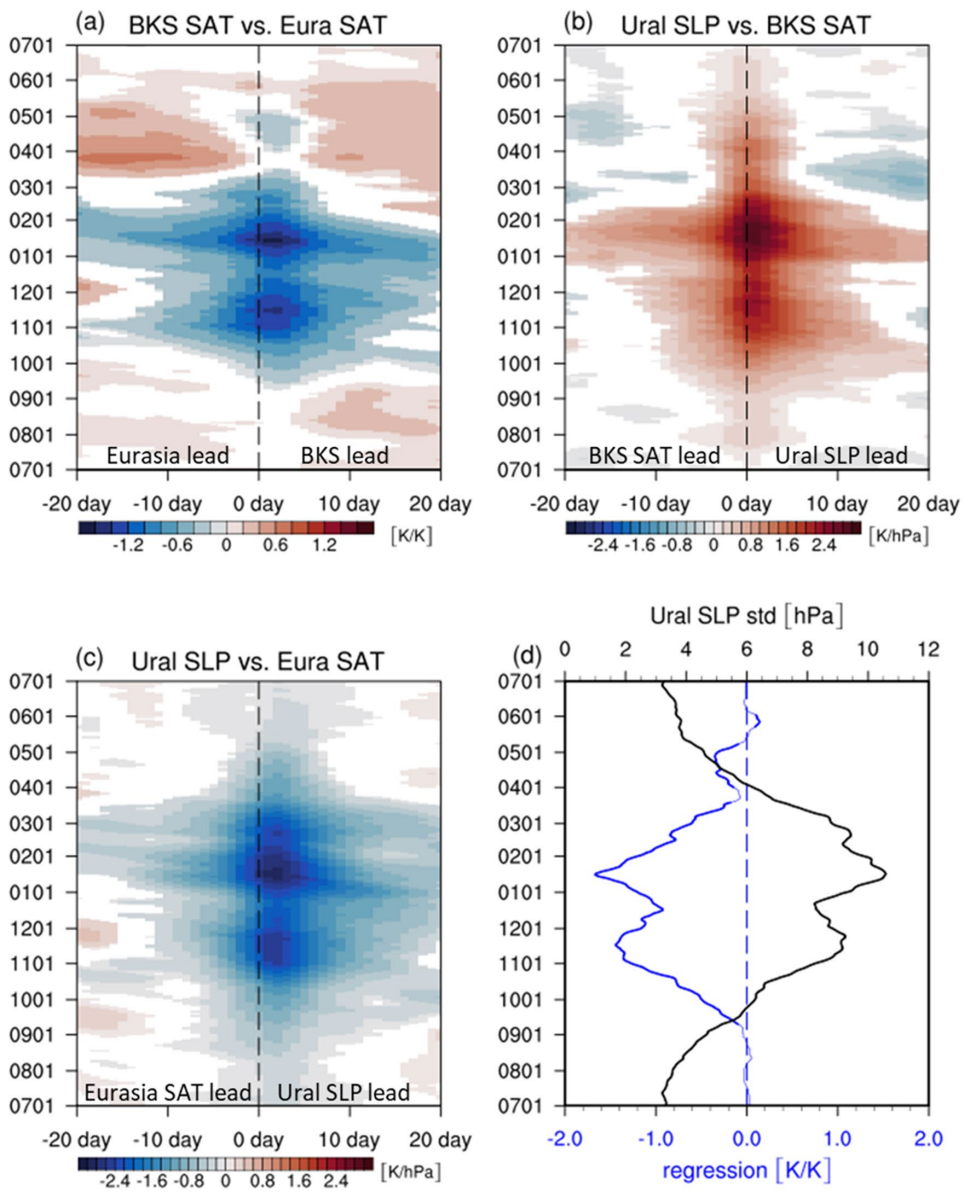
(**a**) Lead–lag regression coefficients of BKS and Eurasian SAT anomalies during the period of 1980–2016. Same as (**a**) but for (**b**) Ural SLP and BKS SAT anomalies, (**c**) for Ural SLP and Eurasian SAT anomalies. The y-axis is the starting day of the 30-day combined time series and the x-axis is the time lag. Only the values that are statistically significant at the 95% confidence level are shaded. (**d**) Regression coefficient (blue) at 2 days in (**a**) and the standard deviation of Ural SLP anomalies (black). Figures were created with the NCAR Command Language 6.6.2 (http://dx.doi.org/10.5065/D6WD3XH5).

Figure 1a shows the time-lagged regression coefficients between the BKS and Eurasian SAT anomalies as a function of the calendar day (see method). A significant negative relationship appears in the cold season (Fig. 1a). Such relationships, which are evident only from November to February, are maintained for over a month, indicating that their time scale is longer than a synoptic scale. This result also suggests that WACE/CAWE relationships is unique only in the boreal winter. Although not shown, the same analyses with other reanalysis data show essentially the same results.

The BKS-Eurasian SAT anomalies exhibit a maximum co-variability at lag 2 days, the former leading the latter (Fig. 1a). The similar lags ranging from 1 to 5 days, depending on the analysis domain, are also found in previous studies^15,18^. This lagged relationship, however, does not indicate that the Arctic sea ice or sea surface temperature condition drives the Eurasian SAT variability. Although the BKS SAT anomaly is closely related to local sea ice and sea surface temperature anomalies, they often lead the Arctic surface condition on a daily time scale^17,18^.

**Figure 2:**
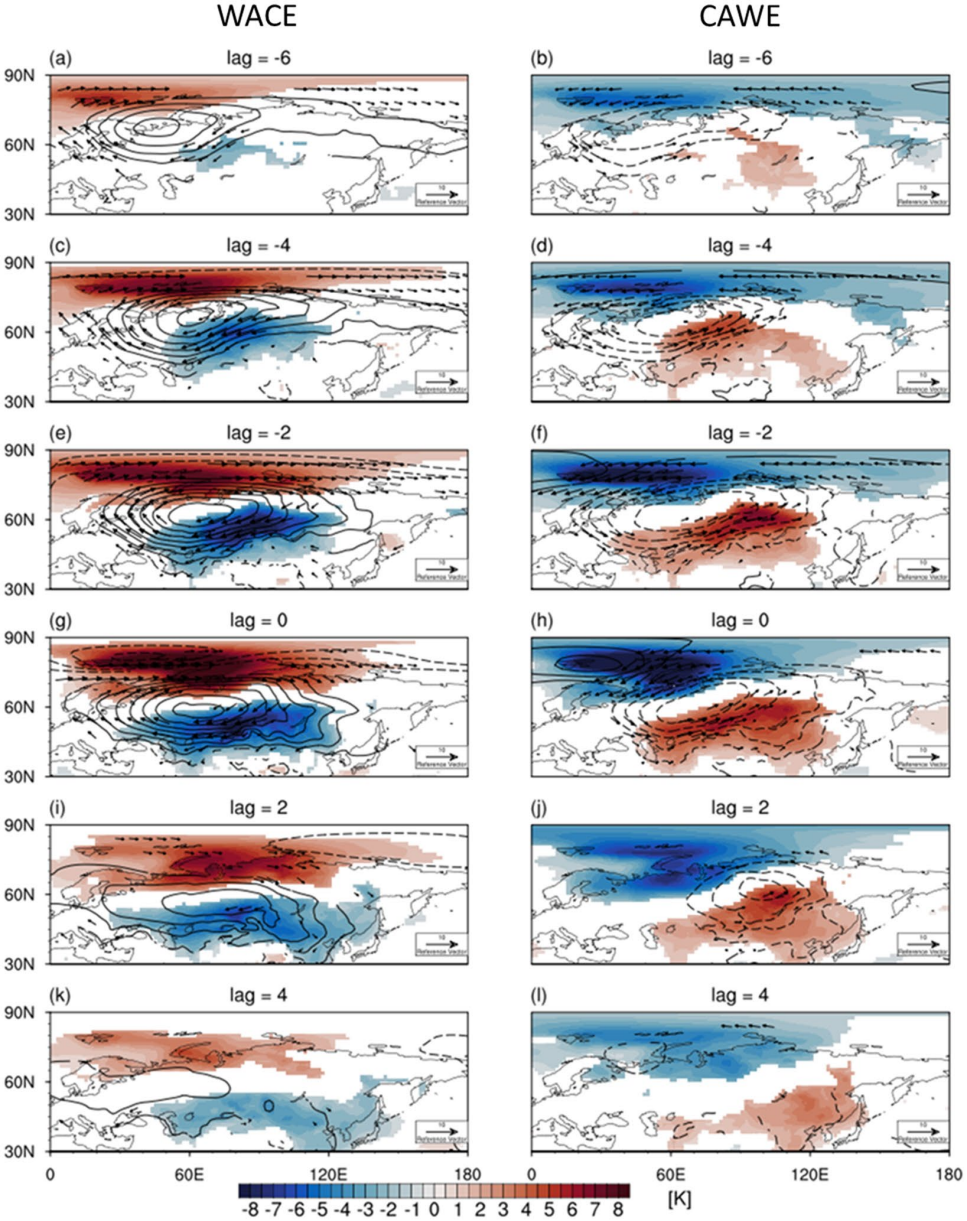
The lead-lag composite mean of SAT (shaded) and SLP (contour) anomalies and wind (vector) anomalies at 850 hPa for (left) WACE and (right) CAWE cases at lags (**a**, **b**) -6 days, (**c**, **d**) -4 days, (**e**, **f**) -2 days, (**g**, **h**) 0 days, (**i**, **j**) 2 days, and (**k**, **l**) 4 days with respect to the local maximum of DI. The contour interval for SLP anomalies is 2 hPa and the negative values are contoured in dashed line. Only the values that are statistically significant at the 95% confidence level are shaded. Figures were created with the NCAR Command Language 6.6.2 (http://dx.doi.org/10.5065/D6WD3XH5).

The lagged relationship is instead largely driven by the SLP anomaly over the Ural region^14,17,18^. Figure 1b,c show strong positive correlations between the BKS SAT and Ural SLP anomalies and negative correlations between the Eurasian SAT and Ural SLP anomalies, respectively. Here the Ural SLP anomaly is calculated over 30°-100°E and 50°-70°N (Fig. S1). While no time lag is observed in Fig. 1b, a 2-day time lag is evident in Fig. 1c. This result suggests that the lagged relationship of the BKS-Eurasian SAT anomalies is caused by the delayed response of the Eurasian SAT to the Ural SLP anomalies. As shown later, such delay occurs as the SLP-induced SAT anomalies expand in time toward the Eurasian domain (Fig. 2). This result is consistent with previous studies^15–18^, showing that the WACE-like SAT pattern is mainly caused by the blocking high over the Ural region rather than the short-term sea ice variability. However, our analysis does not presume the presence of blocking, generalizing the previous findings which were based on blocking composites.

Figure 1a also reveals a bimodal distribution of SAT co-variability. It peaks in November and January with a local minimum in late December. This feature is also found in the regression coefficients of the Ural SLP anomaly (Fig. 1b,c), implying that the double peaks likely result from the circulation anomaly over the Ural region. The regression coefficient between the BKS and Eurasian SAT anomalies at lag 2 days (blue) and the standard deviation of the Ural SLP anomaly (black) are depicted in Fig. 1d for the same period. It becomes clear that the subseasonal variation of the BKS-Eurasian SAT co-variability is strongly influenced by the Ural SLP variability. This result highlights the crucial role of the weather systems over the Ural region in driving dipolar SAT anomalies between the BKS and Eurasia.

The co-variability of the BKS-Eurasian SAT anomalies for WACE and CAWE cases is further illustrated in Fig. 2. Here, only the boreal winter, NDJF, is considered for the case selection. A strong warm anomaly over the BKS and a cold anomaly over the Eurasia are well defined in the WACE cases (left column in Fig. 2). Unlike the BKS SAT anomaly, the Eurasian SAT anomaly expands southeastward in time. This temporal evolution is in agreement with the time lag in regression between the Ural SLP and Eurasian SAT anomalies shown in Fig. 1c.

The anticyclonic anomaly over the Ural region is quasi-stationary and maintained over 10 days, representing the Ural blocking^15,19^. To quantify the contribution of the Ural blocking to the WACE SAT variability, the WACE cases are grouped into those with and without the Ural blocking. Here, the blocking is identified with the Tibaldi-Molteni index^29^, which is defined as a meridional gradient reversal of Z500 from south (${\upphi }_{\mathrm{S}}=40^\circ \mathrm{N}+ \Delta$) to north (${\upphi }_{\mathrm{N}}=80^\circ \mathrm{N}+ \Delta$). The choice of ∆ is from -4.5° to 4.5° in 1.5° increment. The Ural blocking is identified when the gradient reversal persists more than three consecutive days over the longitude band of 30-100ºE. The blocking-related WACE cases are then classified when the Ural blocking is detected within ± 3 days of the WACE date (lag 0). Otherwise, the WACE cases are not related to the Ural blocking.

**Figure 3:**
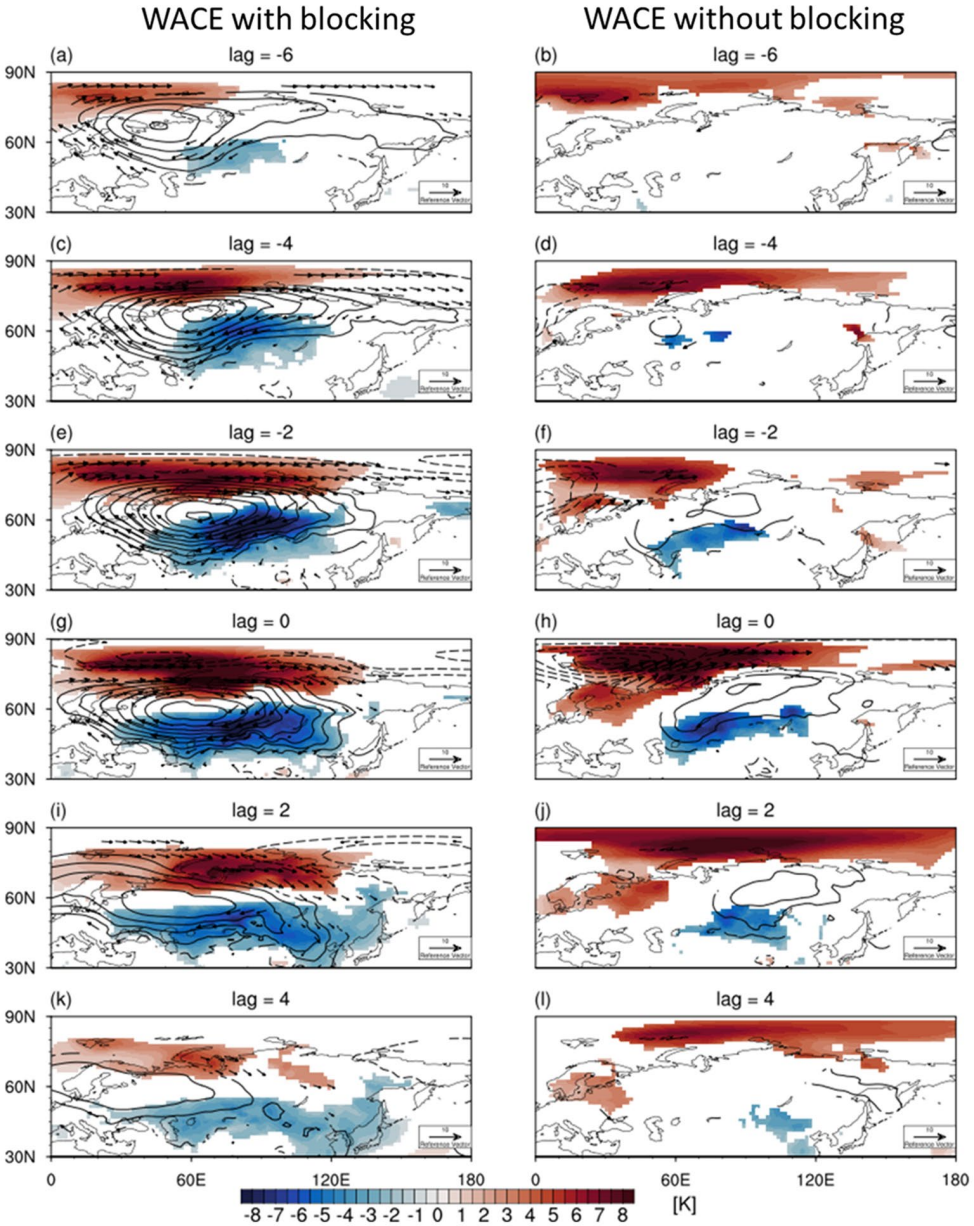
Same as Fig. 2 but for WACE cases (left) with blocking and (right) without blocking. Figures were created with the NCAR Command Language 6.6.2 (http://dx.doi.org/10.5065/D6WD3XH5).

It turns out that 78% of the WACE cases are accompanied with the Ural blocking which slowly moves westward in time (left column of Fig. 3). This result is consistent with the previous studies that suggest the Ural blocking as a key driver of the subseasonal WACE pattern^15,19,24^. However, the other 22% are not directly related to the Ural blocking (right column in Fig. 3). They are instead associated with an eastward-moving anticyclone which is weaker and less persistent than the blocking high.

This result indicates that the Ural blocking is not a necessary condition for the WACE pattern. A well-defined anticyclonic anomaly, regardless of westward or eastward propagation, can result in the WACE pattern. It is noticeable that although the amplitude of SAT anomaly is comparable between the blocking and non-blocking WACE cases, the Ural blocking leads to more persistent anomaly in a broader region over the Eurasia. This implies that the Ural blocking is still an important factor in determining SAT anomalies (compare Fig. 3g,h).

The CAWE cases show a similar result to the WACE cases with an opposite sign (right column in Fig. 2). One distinctive difference from the WACE cases is the propagation of the Ural SLP anomaly. Unlike the quasi-stationary SLP anomaly of the WACE cases due to the mixture of the westward-moving Ural blocking and the eastward-moving transient anticyclone, the SLP anomaly of the CAWE cases migrates eastward through time. Its structure more resembles the SLP anomaly of the non-blocking WACE cases (right column in Fig. 3). This result indicates that the CAWE pattern is mainly determined by the transient cyclonic anomaly over the Ural region.

**Figure 4:**
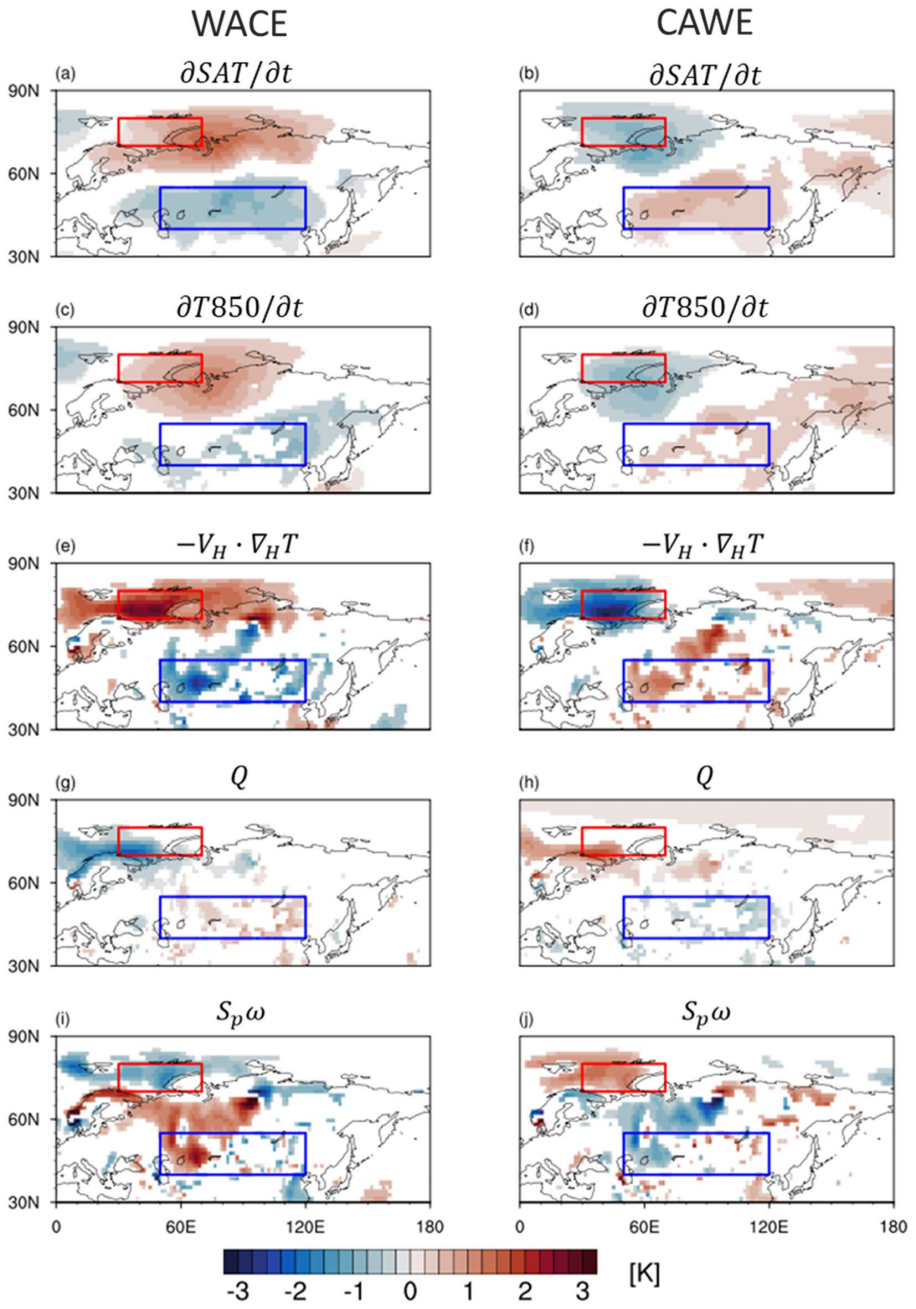
(**a**, **b**) The SAT tendency, (**c**, **d**) T850 tendency, (**e**, **f**) horizontal temperature advection, (**g**, **h**) diabatic heating, (**i**, **j**) adiabatic heating averaged from -5 to 0 days for (left) WACE and (right) CAWE cases. Note that all values are slightly smoothed by applying a nine-point local smoothing once and the underground values are excluded. Only the values that are statistically significant at the 95% confidence level are shaded. Figures were created with the NCAR Command Language 6.6.2 (http://dx.doi.org/10.5065/D6WD3XH5).

**Figure 5:**
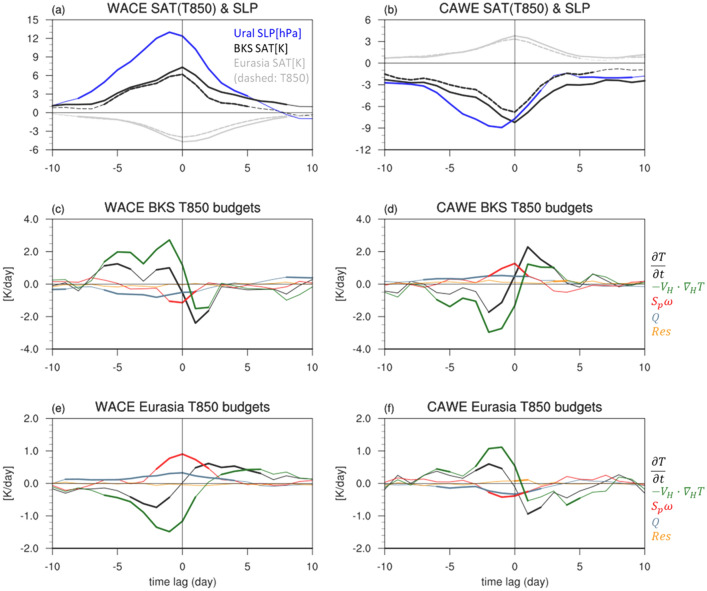
(**a**) Time evolution of lead-lag composites of BKS (black) and Eurasian (gray) SAT (solid line), T850 (dashed line), and Ural SLP (blue) for the WACE cases. Lead-lag composites of temperature tendency and anomalous budget terms in (**c**) BKS and (**e**) Eurasia. (**b**,**d**,**f**) Same as in (**a**,**c**,**e**) but for CAWE cases. The statistically significant values at the 95% confidence level are bolded. Figures were created with the NCAR Command Language 6.6.2 (http://dx.doi.org/10.5065/D6WD3XH5).

To identify the physical processes that determine the WACE and CAWE patterns, temperature budget is computed at the 850-hPa pressure level (see method). The 850-hPa temperature (T850) is used here to minimize the topographic effect over the Eurasia. Figure 4a,b shows the spatial distribution of the SAT tendency, averaged from − 5 to 0 day, for the WACE and CAWE cases. They are well captured by T850 tendency (Fig. 4c,d), justifying the use of T850 instead of SAT in temperature budget analysis (see also Fig. 5a,b).

For the WACE cases, the horizontal temperature advection (Fig. 4e) explains most of the temperature change over the BKS and Eurasia. The diabatic heating shows an opposite sign to the temperature advection (Fig. 4g). The adiabatic heating matches well with the SLP anomalies (Fig. 4i). Downward motion over the anticyclonic anomaly results in the adiabatic warming, while it also accompanies the adiabatic cooling in the northwest and southeast. Here it is important to note that both diabatic (Fig. 4g) and adiabatic heatings (Fig. 4i) *cancel* the advective warming over the BKS and cooling over the Eurasia. This result is in stark contrast to the previous studies which highlighted the role of the diabatic heating especially the downward longwave radiation^15,24^.

Figure 5c,e confirms the results presented in Fig. 4. They show the temporal evolution of BKS and Eurasia T850 anomalies during the growth (− 10 to 0 days) and decay phases (0 to 10 days). As shown in Fig. 4, the horizontal temperature advection (green) dominates the BKS T850 tendency (Fig. 5c). Both diabatic (blue) and adiabatic heatings (red) *cancel* the advective warming. Before the WACE date (lag 0 day), net diabatic heating is negative, and this is statistically significant. The adiabatic cooling appears around lag 0 day, largely cancelling the temperature advection during the WACE date. A similar mechanism holds for the Eurasian T850 tendency (Fig. 5e) with a larger cancellation between the advective cooling and adiabatic warming.

The same analysis is also conducted for the WACE cases with and without blocking (Fig. S2). As shown in Fig. 3, the amplitude of SAT anomalies over BKS is comparable although the Eurasian SAT and Ural SLP anomalies of the blocking WACE cases are much stronger (Fig. S2a,b). This result may imply that temperature anomaly over BKS is only weakly dependent on the presence of the Ural blocking while the Eurasian cold temperature anomaly is strongly influenced by the Ural blocking. It is further found that regardless of the presence of blocking, the temperature advection is the primary process that drives the WACE pattern. Figure 4b,d,f shows the T850 budget for the CAWE cases. Overall results are consistent with the WACE cases. It is concluded that both WACE and CAWE cases are mainly driven by the horizontal temperature advection.

To examine the sensitivity of the result to the choice of the pressure level, the temperature budget at 850 hPa integrated from lag -5 to 0 days is further compared to that at 925 hPa (Fig. S3). The diabatic heating is re-calculated as a residue by subtracting the all terms from the temperature tendency. This allows to mask the missing values over the complex terrain in Eurasia. A small residue in Fig. 5 justifies this approach. During the WACE growth phase from –lag 5 to 0 days, the mechanism holds at both 850 and 925 hPa (Fig. S3a,c). Although the diabatic heating becomes stronger near the surface (e.g. for BKS, − 4.5 K at 850 hPa to − 10.0 K at 925 hPa), while the horizontal advection also becomes stronger (e.g. for BKS, 11.2 K at 850 hPa to 16.7 K at 925 hPa). They are effectively cancelled out. The same result is also found for the CAWE cases (Fig. S3b,d).

## Summary and discussion

A negative relationship between the BKS and Eurasian SAT anomalies is identified on a daily time scale. This relationship is primarily caused by synoptic weather systems over the Ural region. This is consistent with the previous studies that highlighted the role of the Ural blocking^15,17,19^, but is more generalized as not only quasi-stationary blocking highs but also transient weather systems are considered in this study. It is further found that the dipolar SAT relationship is driven not only by the WACE cases but also by the CAWE cases. Both of them are frequently observed from November through February with a local minimum in late December, although what makes a local minimum is not clear.

It is found that the key process by which the Ural SLP anomaly determines temporal evolution of the WACE and CAWE cases is the horizontal temperature advection. Although previous studies often addressed the importance of the downward longwave radiation and surface heat flux especially on warm Arctic, the net radiative heating over the BKS, estimated from ERA-Interim model output, is negative. Its impact is mostly confined in the lower level. Note that the previous studies have considered only one or two terms in temperature budget (e. g., horizontal advection, surface fluxes, or downward long wave radiation)^15,24,25^. In this study, all terms including horizontal advection, adiabatic heating, and diabatic heating, are computed and the cancellation between the horizontal temperature advection and the diabatic heating is highlighted.

The SLP anomaly responsible for the WACE and CAWE cases is not forced by Arctic sea ice or North Atlantic sea surface temperature anomaly (not shown). In this regard, the subseasonal WACE/CAWE patterns differ from the interannual WACE/CAWE patterns. The interannual WACE pattern has often been explained by the planetary wave train induced by anomalous heating over Gulf Stream^30^ or the Arctic sea ice loss^5–8^. Likewise, the CAWE or the negative WACE pattern has been explained by the stationary wave initiated over the North Atlantic^10,31,32^.

On interannual time scale, the WACE pattern has also been related with the stratospheric variability^5,33^. A series of climate model simulations showed that a weak polar vortex, partly resulting from the reduced Arctic sea ice concentration, could lead to cold Eurasia in late winter^5,33–35^. Although the time scale is different, such a possibility is tested here by constructing the probability distribution function of the polar-cap averaged geopotential height anomaly at 50 hPa (Fig. S4). It turns out that the WACE cases in late winter are slightly biased to the negative geopotential height anomaly or weak polar vortex as in the interannual WACE cases (Fig. S4a). This bias is absent in the CAWE cases, indicating a subtle difference between the WACE and CAWE cases.

The WACE and CAWE cases show different decadal variability. When the number of WACE and CAWE cases are counted before and after year 2000, the WACE cases increase from 1.55 to 2.44 cases per year. But the CAWE cases decrease from 2.65 to 0.89 cases per year. This asymmetry is consistent with more frequent Ural blockings^15,17,36^ and weakened Eurasian storm activities in the recent decade^16,37^. It was argued that the reduced meridional temperature gradient, resulting from the Arctic amplification, weakens the local baroclinicity and storm activities^15,36–38^. This provides a favorable condition for more persistent and stationary blockings^15,35,39^, increasing the possibility of more frequent WACE cases. This result suggests that although the daily BKS-Eurasian SAT co-variability is a natural feature, the ratio of the WACE cases to the CAWE cases has changed (and will change) with time in response to the Arctic amplification. To better understand such change, the decadal variability of the WACE and CAWE cases in climate models warrants further investigation.

## Supplementary Information

Supplementary Information 1.
