# Supplementary material for: Subseasonal relationship between Arctic and Eurasian surface air temperature
Source: Sci Rep. 2021 Feb 18;11:4081. doi: 10.1038/s41598-021-83486-5 (PMC7892886; doi:10.1038/s41598-021-83486-5)
Supplement: Supplementary file 1 — Supplementary Information 1. [file 41598_2021_83486_MOESM1_ESM.docx]

Supplementary Information

Subseasonal relationship between Arctic and Eurasian surface air temperature

Hye-Jin Kim^1^, Seok-Woo Son^1*^, Woosok Moon^2,3^, Jong-Seong Kug^4^, and Jaeyoung Hwang^1^

^1^School of Earth and Environmental Sciences, Seoul National University, Seoul, Korea

^2^Department of Mathematics, Stockholm University, Stockholm, Sweden

^3^Nordic Institute for Theoretical Physics (NORDITA), Stockholm, Sweden

^4^Division of Environmental Science and Engineering, Pohang University of Science and Technology (POSTECH), Pohang, Korea

Corresponding author: Seok-Woo Son (seokwooson@snu.ac.kr)

†1 Gwanak-ro, Gwanak-gu, Seoul, 08826, South Korea.


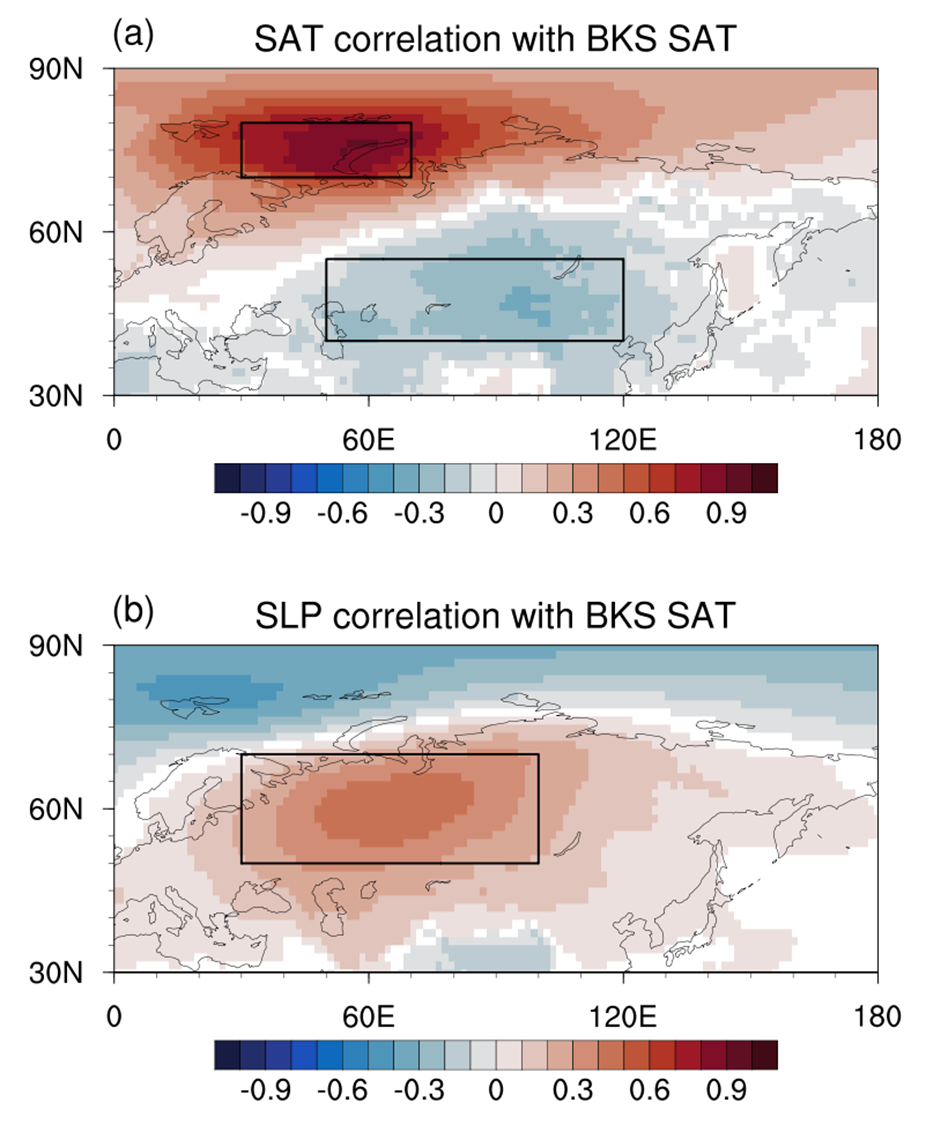


**Figure S1**. Correlation coefficients of (a) SAT and (b) SLP with respect to daily SAT anomalies averaged over the BKS for the period from 1979/1980 to 2016/2017 NDJF. Only the values that are statistically significant at the 95% confidence level are shaded. The analysis domains of the BKS and the Eurasian continent are denoted with boxes in (a). Likewise, the Ural region is denoted with a box in (b). Figures were created with the NCAR Command Language 6.6.2 (http://dx.doi.org/10.5065/D6WD3XH5)

**
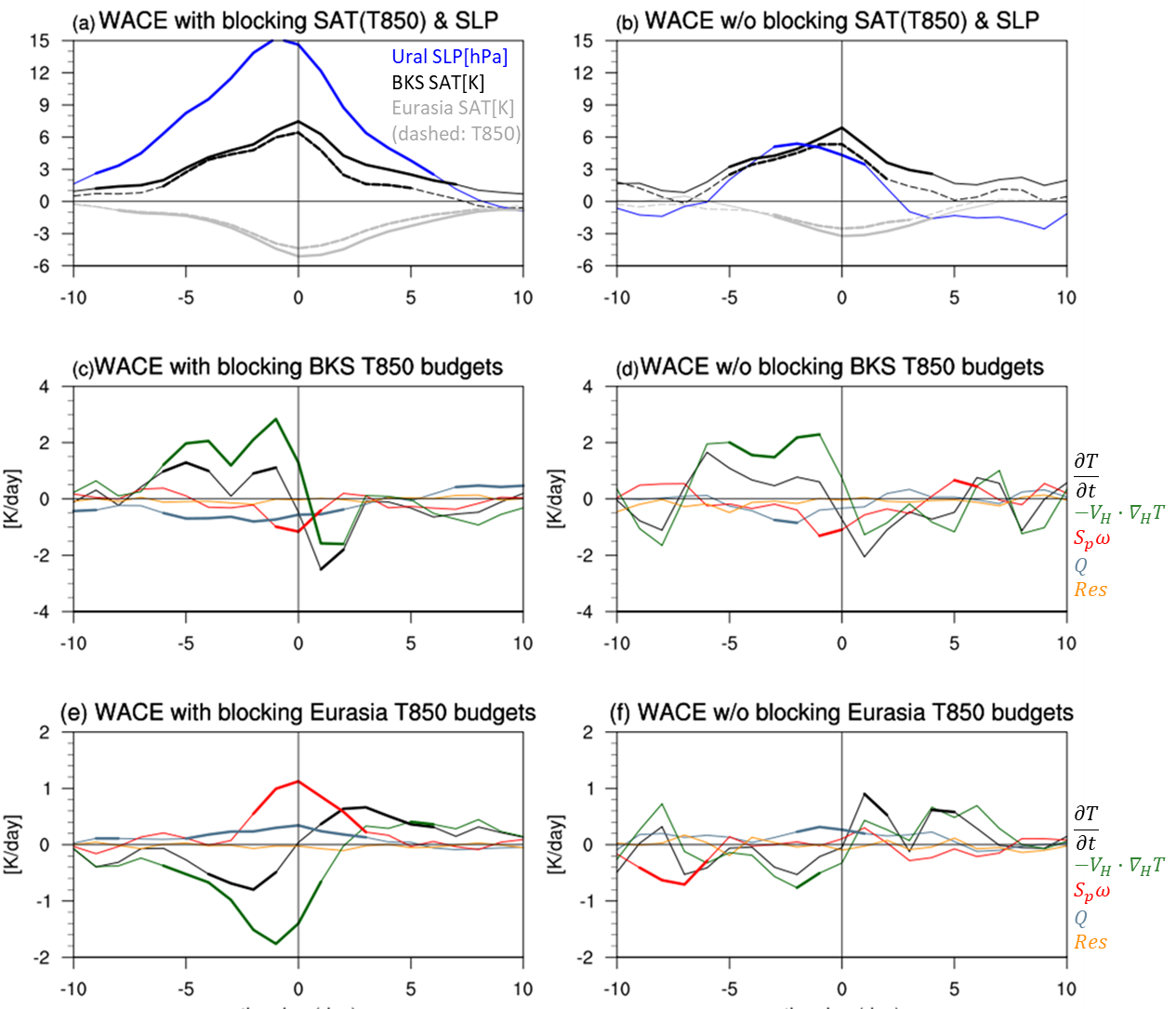
**

**Figure S2**. Same as Fig. 5 but for WACE cases (left) with and (right) without blocking. Figures were created with the NCAR Command Language 6.6.2 (http://dx.doi.org/10.5065/D6WD3XH5)


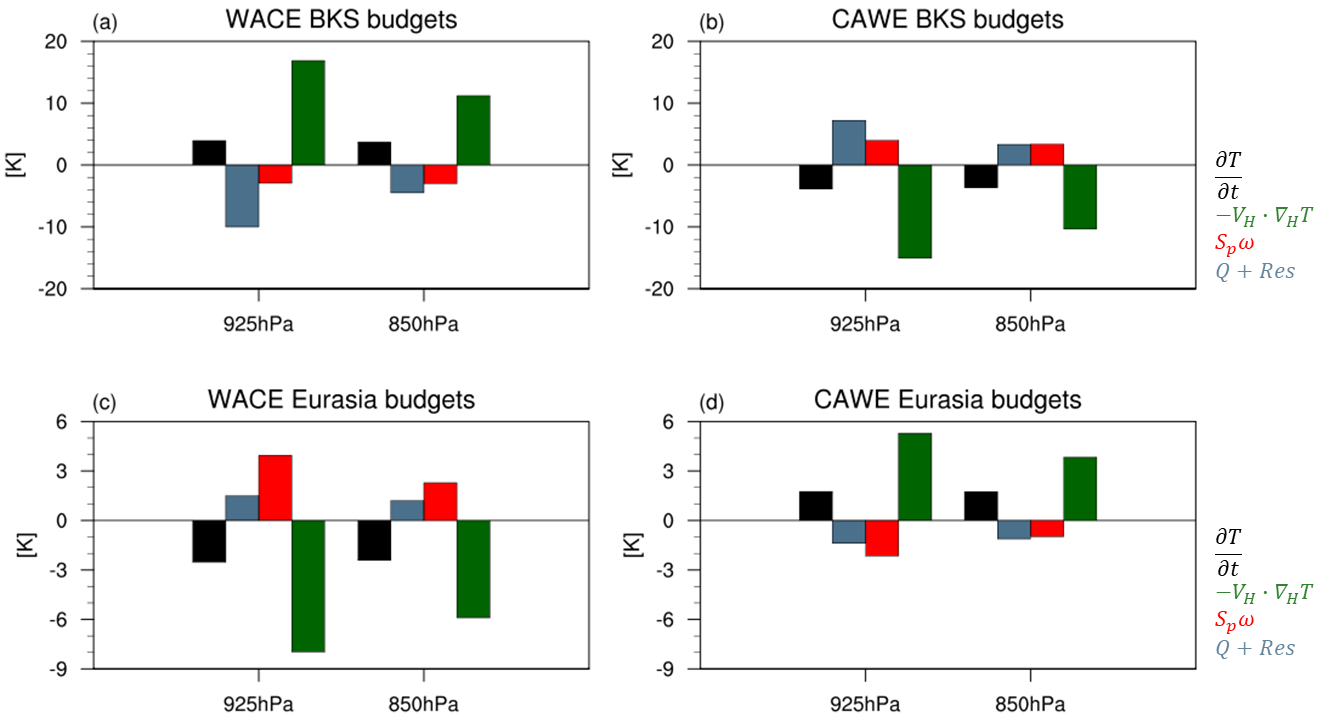


**Figure S3.** The time-integrated temperature tendency and budget terms for lag -5 to 0 days over (a-b) BKS and (c-d) Eurasia for (left) WACE and (right) CAWE cases. Figures were created with the NCAR Command Language 6.6.2 (http://dx.doi.org/10.5065/D6WD3XH5)


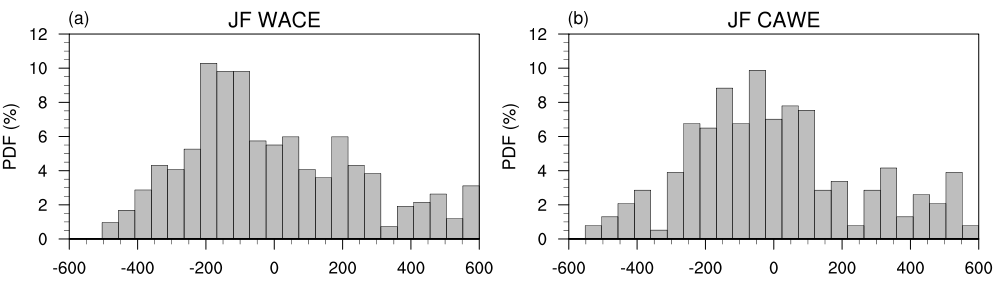


**Figure S4.** Histogram of polar cap (north of 65°N) averaged geopotential height anomaly at 50 hPa for (a) WACE and (b) CAWE cases in January and February. For each WACE or CAWE case, lag -3 to 3 days are considered to obtain the histogram. Figures were created with the NCAR Command Language 6.6.2 (http://dx.doi.org/10.5065/D6WD3XH5)
